# Force-Chain Networks and Particle-Scale Mechanics of Granular Materials Under Low-Confinement Quasi-Static Shear

**DOI:** 10.3390/ma19132696

**Published:** 2026-06-23

**Authors:** Hui Luo, Yangshuai Zheng

**Affiliations:** 1 State Key Laboratory of Geo-Hazard Prevention and Geo-Environment Protection, Chengdu University of Technology, Chengdu 610059, China; huiluo1939@163.com; 2 College of Intelligent Construction and Manufacturing, Sichuan Technology and Business University, Chengdu 611745, China

**Keywords:** granular materials, discrete element method, microstructural characterization, force-chain network, quasi-static shear, bulk solids characterization

## Abstract

Dense granular materials under low confining stress and low shear velocity—conditions relevant to low-pressure powder handling, near-surface transport, and the upper layers of stored bulk solids—remain insufficiently characterized at the microstructural level. We perform three-dimensional discrete element method (DEM) simulations of annular shear of monodisperse glass spheres at σ = 1 kPa and v = 0.01 m/s, corresponding to an inertial number I ≈ 1.06 × 10^−3^ at the quasi-static limit of the dense flow regime. The steady-state friction coefficient stabilizes at μ_ss_ ≈ 0.78, consistent with the quasi-static limit of the μ(I) framework. The solid volume fraction decreases monotonically from φ ≈ 0.50 at the base to φ ≈ 0.35 near the top, while the tangential velocity decays exponentially with depth (decay length δ_s_ ≈ 10 mm). Particle trajectory tracking reveals a sharp kinematic transition near z ≈ 5–6 mm separating a quasi-rigid basal layer (z ≲ 5 mm) from an upper shear-active zone (z ≳ 6 mm). The contact force distribution follows an exponential decay P(f/⟨f⟩) ∝ exp(−β·f/⟨f⟩) with β ≈ 0.45, with strong force chains selectively concentrated in the upper zone. Together, these four microstructural descriptors co-locate within a single transition band, providing quantitative benchmarks for material characterization and constitutive modelling at the lower boundary of dense flow.

## 1. Introduction

Granular materials constitute one of the most widely encountered classes of engineering matter, spanning applications from construction aggregates and pharmaceutical powders to catalyst supports, additively manufactured feedstocks, and bulk solids in storage and conveying systems [1,2]. The mechanical response of these materials under shear loading is central to their processing, handling, and performance, and differs fundamentally from that of conventional solids: deformation is mediated by particle-scale rearrangements, contact-force redistribution, and the formation and rupture of force-chain networks rather than by continuous internal strain. Characterizing these microstructural processes is therefore essential for the rational design of granular-material-based systems and for the development of predictive constitutive models [3,4].

The mechanical regime of a sheared granular material is most compactly described by the dimensionless inertial number $I=\dot{\gamma }d/\sqrt{P/\rho }$, where $\dot{\gamma }$ is the shear rate, d the particle diameter, P the confining pressure, and ρ the particle density [5]. Early theoretical frameworks established the kinetic-theory description of rapid granular flows and the constitutive behaviour of dry cohesionless assemblies in annular shear cells [6,7]. At low I (<10^−3^), sustained frictional contacts dominate and the material responds quasi-statically. At intermediate I (10^−3^ to 10^−1^), inertial and frictional effects coexist, defining the dense flow regime in which the μ(I) rheological framework has been extensively validated [5,8]. At high I (> 10^−1^), grain collisions dominate and the material transitions toward a dilute, gaseous state [9]. The μ(I) framework expresses macroscopic friction and solid fraction as smooth, monotonic functions of I, and has been benchmarked across a broad range of geometries and material systems through both experiments and simulations [5,8,9,10].

Despite the maturity of this framework at moderate-to-high stress levels, the low-confinement end of the dense flow regime—where applied normal stress is of order 1 kPa and shear velocity is of order 0.01 m/s—remains comparatively underexplored. This parameter range is directly relevant to a number of materials-engineering contexts in which granular materials experience low overburden and slow deformation, including the upper layers of stored bulk solids, low-pressure powder handling, near-surface granular transport, and the early stages of shallow geomaterial mobilization [11,12]. In these conditions, the assumptions underlying the dense-flow rheology—well-developed inertial forcing and uniformly distributed contact networks—may not hold, and microstructural heterogeneity is expected to play a more decisive role in governing the material response [13,14].

Previous investigations have addressed individual aspects of low-inertia granular shear in isolation. Velocity profiles and shear localization have been documented in annular and Couette geometries [15,16], and contact force statistics have been characterized in quasi-static assemblies [17,18]. Particle trajectory analysis has revealed layer-dependent vertical oscillations and net migration [19], while solid fraction profiles have been linked to the competition between shear-induced dilatancy and gravitational compaction [10]. Non-local rheological models incorporating spatial correlations of plastic activity have been proposed to capture deviations from the local μ(I) description near jamming [20,21,22]. What remains lacking, however, is a unified microstructural characterization that simultaneously resolves these descriptors within a single low-confinement system and identifies how they are spatially coupled rather than independently governed.

While our recent work [23,24] has examined the effects of overburden and shear velocity on the macroscopic flow behaviour of such systems, the microstructural origin of the observed flow patterns—particularly, the spatial coupling between force-chain topology, packing variability, and particle-scale kinematics—remains uncharacterized. In this study, we perform three-dimensional discrete element method (DEM) simulations of a dense monodisperse granular material under annular shear at σ = 1 kPa and v = 0.01 m/s, corresponding to a nominal inertial number I ≈ 1.06 × 10^−3^ at the quasi-static limit of the dense flow regime, and provide a coupled particle-scale characterization of its mechanical behaviour. We concurrently resolve the macroscopic friction response, height-resolved tangential velocity and fluctuation profiles, solid volume fraction distribution, Lagrangian particle trajectories, and contact force-chain network topology. By examining these microstructural descriptors jointly rather than in isolation, we identify the spatial coupling that defines the material state in the low-confinement dense flow regime and provide quantitative benchmarks for constitutive model validation and for the engineering characterization of granular materials at the lower boundary of dense flow behaviour. The novelty of this work lies not in the individual phenomena—each of which is well established—but in demonstrating that, at the low-confinement quasi-static limit, the kinematic, packing, and force-network descriptors transition within a single narrow height band and are thus coupled manifestations of one stratified state. This coupled, co-located characterization, together with the quantitative benchmarks it yields (μ_ss_, β, the kinematic threshold, and the φ(z) profile), advances existing DEM studies—which have resolved these descriptors separately and at higher confinement—and provides specific targets for constitutive-model validation at the lower boundary of dense flow.

## 2. Simulation Methodology

### 2.1. Model Geometry and Boundary Conditions

The dense granular column consists of monodisperse glass spheres of diameter d = 1 mm and density ρ = 2500 kg/m^3^, with an initial bed height H = 15 mm. Shear is imposed by rotating the upper platen at a constant tangential velocity v = 0.01 m/s while maintaining a normal stress σ = 1 kPa on the upper boundary. The corresponding mean shear rate is $\dot{\gamma }$ = v/H = 0.667 s^−1^, and the inertial number, defined as $I=\dot{\gamma }d/\sqrt{P/\rho }$, takes the value I ≈ 1.06 × 10^−3^. It should be emphasized that this value is a nominal, bulk-averaged inertial number evaluated from the imposed boundary conditions ($\dot{\gamma }$ = v/H and σ); the local inertial number I(z) is a depth-dependent field, since the local shear rate and confining pressure vary with height (Section 3), whereas the particle density ρ and diameter d remain constant. The nominal value thus characterizes the overall flow regime rather than the state of any individual layer. The system thus operates near the lower bound of the dense flow regime, where frictional contacts dominate the mechanical response and the assumptions underlying the μ(I) rheological framework are tested at the quasi-static limit [5,8].

**Figure 1:**
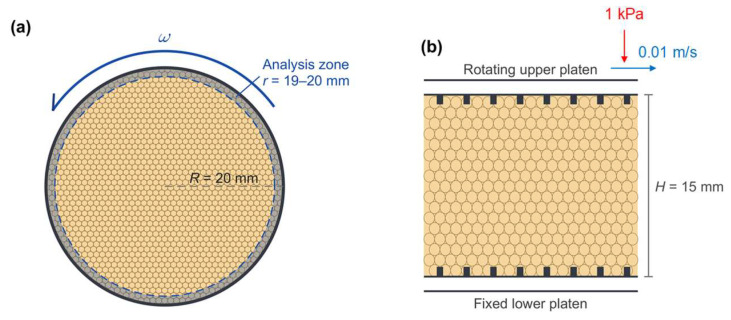
Schematic of the annular shear cell. (**a**) Plan view showing the granular assembly (R = 20 mm) and the analysis annulus r = 19–20 mm (blue shading); ω indicates the rotation direction. (**b**) Cross-section of the granular layer (H = 15 mm) confined between two toothed platens. A normal stress σ = 1 kPa is applied to the upper platen, which is driven at v = 0.01 m/s at the outer radius.

The shear cell (Figure 1) is a hollow cylinder of outer radius R = 20 mm bounded by a smooth, rigid lateral wall that confines the grains radially without exerting tangential drag. The annular geometry was adopted to reproduce the boundary conditions of the laboratory ring-shear apparatus used in our companion experiments [23], rather than for computational convenience; the chosen ratios (R = 20d, H = 15d) fall within the range of comparable DEM ring-shear studies. The radial velocity gradient intrinsic to this configuration is why all kinematic and force statistics are restricted to the outermost annulus (r = 19–20 mm), which spans many particle diameters along the flow direction and best approximates ideal Couette flow, thereby limiting the influence of the finite R/d ratio. The upper and lower platens are textured with 1 mm tooth elements to prevent wall slip and ensure that the imposed boundary motion is fully transferred to internal shear. The lower platen is held fixed; the upper platen rotates at a constant tangential speed v = 0.01 m/s at r = R. The normal stress σ is maintained through a servo-controlled vertical adjustment of the upper platen, which holds the integrated reaction force at σ·πR^2^ while allowing the column to dilate or compact freely as the microstructure evolves.

The shear protocol consists of a 0.5 s consolidation phase, during which the assembly equilibrates under the target normal stress, followed by 8 s of continuous shear. The quasi-steady regime is identified from the concurrent stabilization of the friction coefficient and the upper-platen axial position, and is reached at t ≈ 2 s. All analyses draw on the subsequent 6 s window, sampled every 0.5 s, with spatial fields constructed on a 1 mm vertical binning grid. Kinematic and force statistics were evaluated within the outermost annulus r = 19–20 mm, where the tangential velocity field is most uniform and best approximates the imposed Couette shear [15,16].

### 2.2. Contact Model and Material Parameters

Interparticle and particle–wall interactions were resolved using a nonlinear elasto-frictional contact model in LIGGGHTS [25]. The normal response follows Hertzian elasticity, with the contact stiffness derived from the grains’ elastic moduli and Poisson’s ratio, and the contact force scaling as δ_n_^3/2^. The tangential response follows the Mindlin no-slip solution up to the Coulomb limit μ|F_n_|, beyond which sliding occurs [26,27]. Normal dissipation is introduced through a velocity-dependent viscous term calibrated to a restitution coefficient e = 0.938 [28]. To suppress the unrealistically free rotation of ideal spheres and to represent the angularity and surface roughness of real grains, a linear angular spring at each contact resists relative rotation [29,30]. Without this term, perfectly smooth spheres rotate unphysically freely and underestimate the shear strength; the adopted coefficients (μ_r_ = μ_rw_ = 0.1) lie within the range established in those calibrations [29,30]. The integration time step was set to Δt = 0.3 t_R_, where t_R_ is the Rayleigh time of the assembly; this value lies within the range of 0.2–0.4 t_R_ established as appropriate for solid-particle DEM simulations [31] and, since t_R_ is a conservative estimate of the contact oscillation period, fully resolves the elastic contact response at each contact while ensuring numerical stability without artificial damping [32]. All material parameters are listed in Table 1. The intrinsic material properties (ρ, G, ν, e) were fixed at established literature values for monodisperse glass spheres, and the friction and rolling-resistance coefficients were adopted from published DEM studies of glass-sphere assemblies [28,29,30]. Rather than being calibrated against bespoke experiments, the adequacy of this parameter set is confirmed a posteriori by the agreement of the simulated steady-state friction coefficient and velocity-profile geometry with our companion ring-shear experiments (Section 4).

### 2.3. Force-Chain and Trajectory Analysis

For microstructural characterization, the contact force network was extracted from the simulation output at each recording instant during the quasi-steady phase, with each contact carrying both a normal force F_n_ and a tangential force F_t_ [17,18]. To resolve the spatial organization of stress transmission, the granular column was discretized into horizontal layers of thickness 1 mm, and contact forces within each layer were normalized by the layer-averaged mean normal force $\left\langle F_{n}\right\rangle$ to remove the effect of depth-dependent loading. Contacts satisfying Fn > $\left\langle F_{n}\right\rangle$ were classified as strong force chains, following the standard weak/strong partition of contact-force networks established by Radjai et al. [18] and widely adopted thereafter [17,33,34], rather than an arbitrary choice. The mean contact force $\left\langle F_{n}\right\rangle$ and the number of strong-chain contacts N_c_ were computed for each layer to construct vertical profiles of the contact network topology. For statistical characterization, the probability density function of normalized contact force, P(f/⟨f⟩), was computed by logarithmic binning over the full ensemble of contacts within the quasi-steady phase and fitted with the exponential form P(f/⟨f⟩) ∝ exp(−β·f/⟨f⟩), where the decay exponent β quantifies the heavy-tailed character of the force distribution [18,33]. Only the tail region f/⟨f⟩ ≥ 1 was used for parameter estimation, since the sub-mean range f/⟨f⟩ < 1 is governed by the geometric distribution of weak contacts and is known to deviate from exponential behaviour [18,34].

To complement the layer-averaged characterization of velocity and contact-force fields, the motion of individual particles was tracked throughout the quasi-steady shear phase. Six representative tracer particles were selected from prescribed initial heights spanning the full bed depth (z_0_ ≈ 0.5, 3.0, 6.0, 9.0, 12.0, and 14.5 mm), corresponding to layers within the basal, transitional, and shear-active zones of the granular column. The selected particles were located within the outer annular region (r = 19–20 mm) at the start of the quasi-steady phase to ensure that they reside in the region of maximum imposed shear and remain accessible to direct comparison with experimental PIV measurements obtained at the transparent sidewall in related ring-shear studies [35]. For each tracer, the three-dimensional position (x, y, z) was recorded at every output interval, from which the velocity magnitude |v|, radial coordinate r(t), angular velocity ω(t), and vertical position z(t) were derived. To test robustness against discrete symmetry breaking, an ensemble of n = 10 particles within a ±0.5 mm shell around each reference height (60 tracers) was additionally sampled, and the height-resolved kinematics averaged as mean ± 1σ. This trajectory-tracking framework resolves features that are inaccessible to layer-averaged descriptors—including individual particle vertical migration, oscillatory excursions, and the persistence of frozen contact configurations in the basal layer—and complements the layer-averaged velocity and force-network analyses described above.

## 3. Simulation Results

### 3.1. Macroscopic Friction Coefficient and Axial Displacement

**Figure 2:**
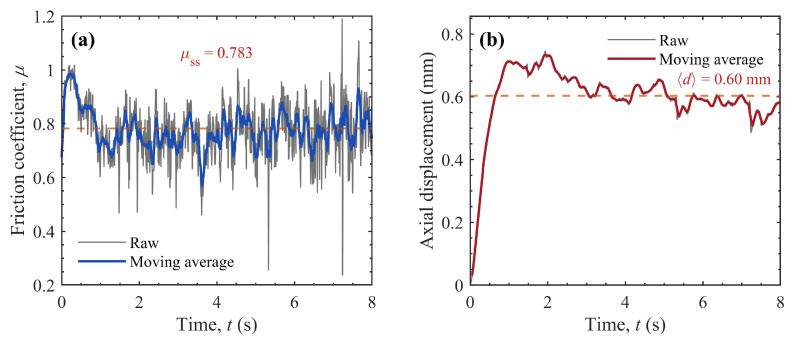
Temporal evolution of (**a**) friction coefficient μ and (**b**) axial displacement of the upper platen under quasi-steady shear (σ = 1 kPa, v = 0.01 m/s). Grey lines: raw signals; coloured lines: moving-average smoothed results. Both signals stabilize at t ≈ 2 s.

Upon the onset of shear, the friction coefficient rises sharply to a transient peak near 1.0 within the first 0.3 s, reflecting rapid mobilization of the interparticle contact network. It subsequently relaxes and stabilizes at a quasi-steady mean value of μ_ss_ ≈ 0.783 (Figure 2a), with persistent fluctuations of amplitude Δμ ≈ 0.1–0.15 that reflect the intermittent contact dynamics characteristic of I ≈ 10^−3^. The axial displacement of the upper platen (Figure 2b) undergoes rapid initial dilation of approximately 0.7 mm within the first 1–2 s, followed by gradual compaction to a quasi-steady displacement of approximately 0.6 mm. Averaged over the quasi-steady window (t > 2 s), the axial displacement is ⟨d⟩ ≈ 0.6 mm (horizontal line in Figure 2b); the small residual drift over the subsequent 6 s confirms that compaction has effectively ceased and is negligible relative to the initial ~0.7 mm dilation, supporting the onset of the quasi-steady regime at t ≈ 2 s. This two-stage response—rapid dilation followed by partial compaction—is consistent with the classical Reynolds dilatancy mechanism under constant normal stress [36]. The concurrent stabilization of both μ and axial displacement at t ≈ 2 s defines the onset of the quasi-steady regime adopted as the reference state for all subsequent analyses.

### 3.2. Tangential Velocity Profile and Velocity Fluctuation

**Figure 3:**
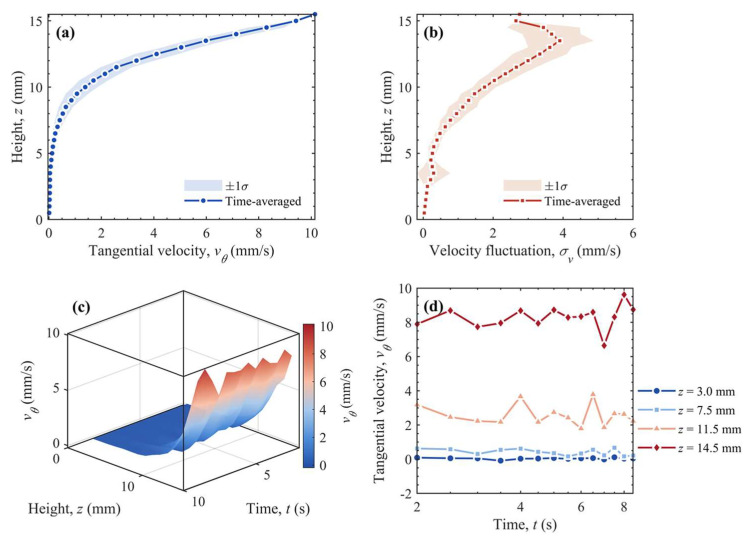
Tangential velocity field under quasi-steady shear. (**a**) Time-averaged mean tangential velocity v_θ_ and (**b**) velocity fluctuation σ_v_ profiles along bed height (markers: time-averaged mean; shaded band: ±1σ envelope over N = 14 snapshots). (**c**) Three-dimensional surface of v_θ_ as a function of height and time. (**d**) Time series of v_θ_ at four representative heights.

The mean tangential velocity profile (Figure 3a) rises monotonically from zero at the base to the imposed boundary velocity at the top, exhibiting a strongly nonlinear, exponential-like decay with depth. Fitting v_θ_(z) = v_0_·exp[−(H − z)/δ] yields a shear band thickness δ_s_ ≈ 10 mm, defined as the depth over which the tangential velocity decays to 1% of its boundary value. This 1-of-boundary-velocity threshold follows the convention used for granular flowing layers by Wang et al. [37], itself adopted by analogy with the turbulent boundary-layer thickness (99% of the free-stream velocity); it is preferred here over linear-extrapolation-to-zero [38] because the present exponential-type profile lacks a distinct linear segment, which would render the latter ambiguous. The lower edge of this band at z ≈ 5 mm coincides with the kinematic threshold identified by particle tracking (Section 3.4), below which the assembly remains essentially quiescent. This pronounced localization is a signature of the low-confinement quasi-static regime, in which the sparse contact network cannot efficiently transmit shear stress to the deeper layers [15,35,39,40]. A preliminary height series (H = 5–20 mm) indicates that the shear-band thickness is essentially invariant for samples tall enough to contain the band (H ≳15 mm), whereas thin samples (H ≈ 5 mm) develop a qualitatively different, near-linear profile. A systematic study of this height dependence is left to future work.

The velocity fluctuation profile (Figure 3b) displays a markedly different spatial structure. The velocity fluctuation σ_v_ is near zero at the base, rises monotonically with height, reaches a broad maximum of approximately 4 mm/s in the range z = 12–14 mm, and declines slightly toward the uppermost layer. The peak location coincides with the region of steepest mean velocity gradient, indicating that fluctuation intensity—an indicator of granular temperature [41,42]—is generated primarily by shear-induced interparticle collisions within the localized shear band. The slight σ_v_ reduction at z ≈ 15 mm is attributed to geometric trapping of particles within the grooves of the toothed driving boundary, which suppresses their lateral fluctuating motion relative to the more mobile particles immediately beneath.

The spatiotemporal evolution in Figure 3c shows that the localized shear structure persists throughout the entire observation window, with no detectable migration of the shear band. This temporal robustness is quantitatively confirmed by the time series at four representative heights (Figure 3d): velocities at z = 3.0 and 7.5 mm remain essentially zero, while those at z = 11.5 and 14.5 mm fluctuate about well-defined mean values of approximately 2.7 and 8.0 mm/s, respectively, with relative fluctuation amplitude increasing from < 5% to ~15% over this height range.

### 3.3. Solid Volume Fraction

**Figure 4:**
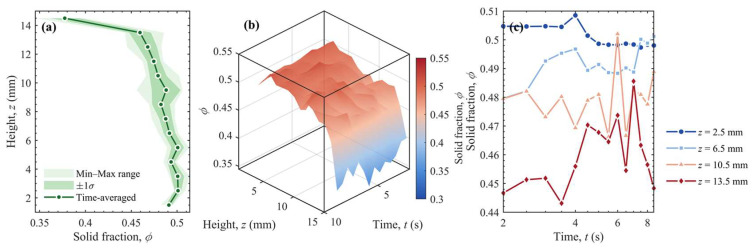
Solid volume fraction φ under quasi-steady shear. (**a**) Time-averaged profile along bed height (markers: mean; dark band: ±1σ; light band: full Min–Max range). (**b**) Three-dimensional surface of φ as a function of height and time. (**c**) Time series of φ at four representative heights.

The time-averaged solid volume fraction profile (Figure 4a) decreases monotonically with bed height, falling from φ ≈ 0.50 at the base to φ ≈ 0.35–0.38 near the free surface. This monotonic decay differs markedly from the bell-shaped profiles reported for intermediate- and high-inertia granular flows, in which shear-induced mid-bed dilation competes with basal compaction to produce a pronounced central density peak [10]. In the present quasi-static regime, the applied normal stress of 1 kPa is insufficient to generate the strong mid-bed compaction required to overcome gravitational settling, so the column equilibrates toward a gravitationally stratified packing in which the densest layers reside at the base of the bed. The vertical gradient ∂φ/∂z becomes pronounced above z ≈ 6 mm, where the looser upper layers accommodate the bulk of the dilation associated with shear localization. Centrifugal effects on the radial packing structure are negligible in this regime: at r = 20 mm and v = 0.01 m/s the angular velocity is ω ≈ 0.5 rad/s, giving a centrifugal acceleration ω^2^r ≈ 5 × 10^−3^ m/s^2^, about three orders of magnitude below gravity. Consistent with this estimate, the solid-fraction field shows no significant radial gradient across the cell, and particles are not systematically displaced outwards.

Temporal variability follows a complementary spatial pattern. Across the lower bed (z < 6 mm), φ remains tightly clustered around its mean (Δφ ≲ 0.01), confirming that the basal layer behaves as a quasi-rigid plug. In the mid-to-upper region (z = 8–14 mm), by contrast, φ fluctuates by Δφ ≈ 0.03–0.05 between successive time steps, and the ±1σ band widens accordingly. This zone of enhanced packing variability coincides spatially with the velocity-fluctuation peak identified in Section 3.2, indicating that transient rearrangements of the contact network and intermittent kinematic excitation occupy the same height band. The three-dimensional evolution surface (Figure 4b) and the time series at four representative heights (Figure 4c) further demonstrate that, despite these local fluctuations, the solid fraction field reaches statistical stationarity after t ≈ 2 s, consistent with the onset of steady-state shear reported throughout the analysis.

### 3.4. Individual Particle Trajectories and Vertical Migration

**Figure 5:**
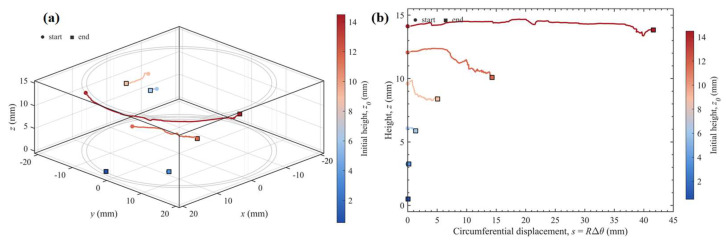
Lagrangian trajectories of six tracer particles under quasi-steady shear (t = 0.5–5.0 s); colour encodes initial height z_0_. (**a**) Three-dimensional trajectories; grey rings mark the analysis region (r = 19–20 mm); filled circles and squares denote start and end points. (**b**) Unwrapped (s, z) view, with the horizontal axis the circumferential displacement s = RΔθ of each tracer relative to its start. Upper tracers (z_0_ ≳ 9 mm) sweep large circumferential distances with marked vertical excursions, whereas basal tracers (z_0_ ≲ 6 mm) remain near s ≈ 0 at fixed height.

**Figure 6:**
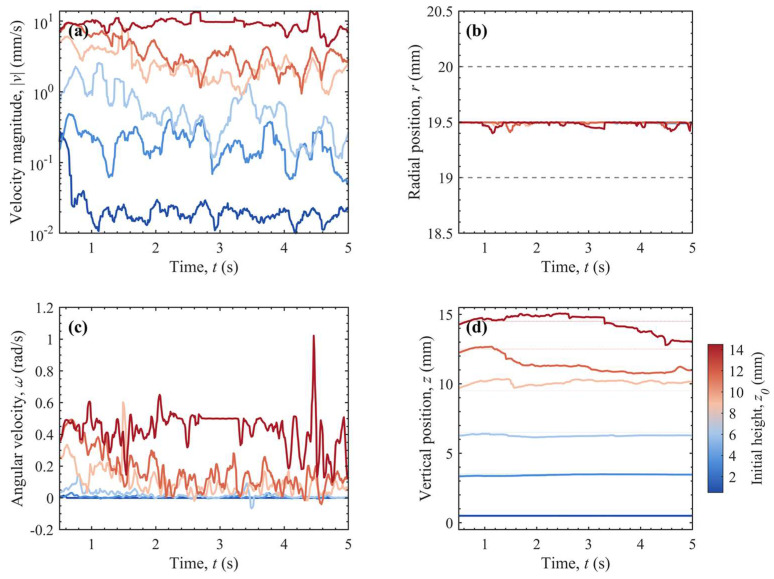
Kinematic time series of six tracer particles. (**a**) Velocity magnitude |v| (log scale). (**b**) Radial position r(t); dashed lines mark the analysis region boundaries. (**c**) Angular velocity ω(t). (**d**) Vertical position z(t); dotted lines indicate initial heights z_0_. Colour encodes z_0_ as in Figure 5.

The three-dimensional trajectories of six representative tracer particles (Figure 5) reveal a sharply stratified kinematic structure: particles originating above z_0_ ≈ 6 mm trace extended arc-like paths within the upper annular region, while particles originating below this height remain effectively frozen throughout the 4.5 s observation window. The kinematic time series in Figure 6 quantifies this stratification. The velocity magnitude (Figure 6a) confirms that upper particles (z_0_ = 14.5 mm) sustain velocities of 5–10 mm/s with clear oscillatory fluctuations on time scales of 0.5–1.0 s, while basal particles (z_0_ = 0.5 mm) maintain velocities below 0.05 mm/s. All particles remain confined within the outer annular zone (Figure 6b), with radial fluctuations of order Δr ≈ 0.5–1.0 mm for upper particles. Tracers initiated at inner radii exhibit the same behaviour as those in the outer annulus, with radial excursions of order one particle diameter and no systematic outward drift, confirming that restricting the reported statistics to the outer annulus r = 19–20 mm does not bias the kinematic results. Angular velocities (Figure 6c) decrease monotonically with initial height, with upper particles sustaining mean values of 0.3–0.5 rad/s and lower particles near zero. Most significantly, the vertical position trajectories (Figure 6d) reveal persistent, large-amplitude vertical oscillations in the upper layers (excursion amplitudes 1–2 mm) alongside a gradual net downward drift for the uppermost tracer, while basal particles remain essentially stationary. The spatial pattern of vertical fluctuation amplitude is consistent with the σ_v_ profile of Figure 3b, reinforcing that the active shear zone coincides with the region of maximum particle agitation. The unwrapped (s, z) representation (Figure 5b), with the horizontal axis giving the circumferential displacement s = RΔθ, makes this stratification directly visible: tracers above z_0_ ≈ 9 mm sweep tens of millimetres circumferentially, whereas those below z_0_ ≈ 6 mm remain near s ≈ 0.

**Figure 7:**
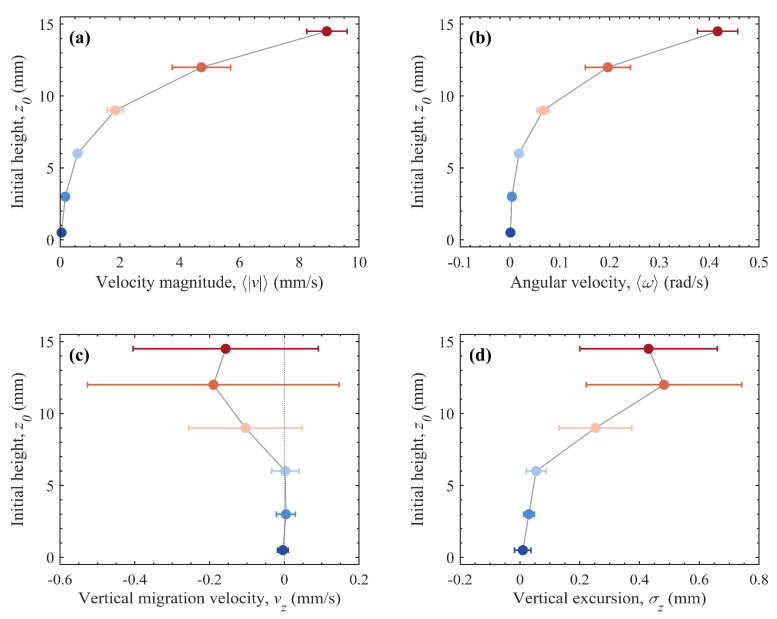
Ensemble-averaged tracer kinematics (n = 10 per height, ±0.5 mm shell, r = 19–20 mm) vs. initial height *z_0_*: (**a**) velocity magnitude ⟨|v|⟩, (**b**) angular velocity ⟨ω⟩, (**c**) net vertical migration velocity *v_z_* (dotted line: *v_z_* = 0), (**d**) vertical-excursion amplitude *σ_z_*. Marker colour encodes initial height z_0_ (blue: basal tracers; red: upper tracers), consistent with Figure 5.

The ensemble-averaged kinematics (n = 10 per height; Figure 7) confirm the stratified mobility as a robust feature: ⟨|v|⟩, ⟨ω⟩, and the excursion amplitude *σ_z_* all rise monotonically with *z_0_* and increase sharply between *z_0_* = 6 and 9 mm, with small inter-particle scatter in the active zone (e.g., ⟨|v|⟩ = 8.9 ± 0.7 mm/s at *z_0_* = 14.5 mm). The upper layers show a weak net downward drift (*v_z_* ≈ −0.1 to −0.2 mm/s for *z_0_* ≳ 9 mm) with large scatter, whereas basal particles (*z_0_* ≲ 6 mm) barely migrate.

### 3.5. Force-Chain Network and Contact Force Statistics

**Figure 8:**
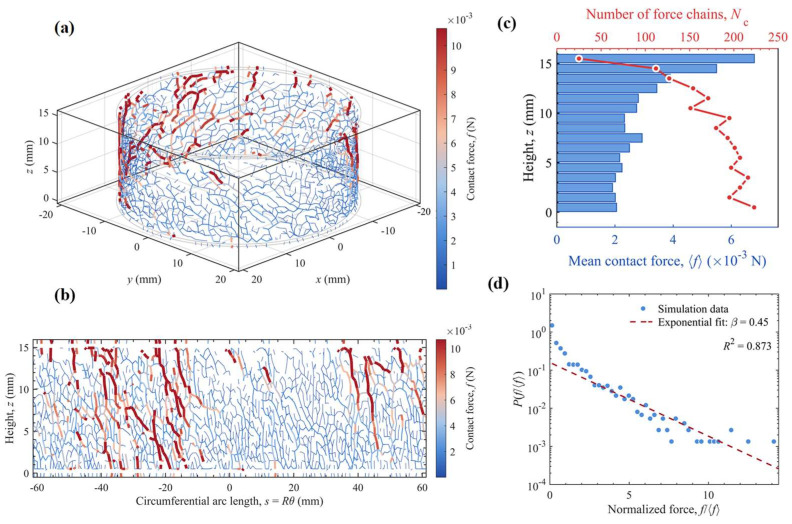
Force-chain network and contact-force statistics under quasi-steady shear. (**a**) Three-dimensional contact-force network in the outer annular region; colour and line width encode contact-force magnitude (upper bound at the 95th percentile). (**b**) Unwrapped (s, z) view of the same network, with circumferential arc length s = Rθ on the horizontal axis at full panel width, showing the selective concentration of strong chains above z ≈ 6 mm. (**c**) Vertical profiles of mean contact force ⟨f⟩ (bars, lower axis) and number of force chains per layer N_c_ (markers, upper axis). (**d**) Probability density function of normalized contact force f/⟨f⟩ on a semi-logarithmic axis; dashed curve: exponential fit with β = 0.45 (R^2^ = 0.873).

The three-dimensional visualization of the contact force network within the outer annular region (Figure 8a) reveals a heterogeneous force structure: a dense background of weak contacts spans the full bed height, while strong, thick chains progressively concentrate above z ≈ 6 mm and become particularly prominent in the uppermost layers (z > 10 mm), in close correspondence with the shear-active zone identified kinematically [17]. The same network unrolled onto the (s, z) plane (Figure 8b) confirms this organization without perspective foreshortening: thick strong chains are sparse near the base and progressively dominate the upper layers (z > 6 mm), while a dense web of weak contacts spans the full height.

The vertical profiles in Figure 8c quantify this spatial organization. Mean contact force increases approximately threefold from the base to the top of the bed, while the number of force chains per layer decreases from a mid-bed maximum to a near-top minimum. This inversion—fewer but stronger chains in the upper layers—indicates that a small subset of persistent strong contacts bears a disproportionate share of the total contact load in the shear-active zone, a microstructural signature of intermittent, heterogeneous stress transmission characteristic of the low-confinement regime.

The strong-force tail is reasonably described to leading order by an exponential, P(f/⟨f⟩) = A·exp(−β·f/⟨f⟩), with β ≈0.45 (R^2^ = 0.873) (Figure 8d). Fitting this tail with exponential, stretched-exponential, and power-law forms yields comparable quality (R^2^ = 0.873, 0.929 and 0.920, respectively); the exponential is retained as the conventional, most parsimonious description of the strong-force network [18,34,43], the alternatives offering only a marginal, statistically non-robust improvement at the noisy far tail. The decay exponent substantially below unity implies a heavy tail in which forces well above the mean are comparatively common, consistent with persistent, long-lived force chains in the quasi-static regime [18,34]. The heavy tail is directly reflected in the strong chains visible in Figure 8a, confirming the mutual consistency of the statistical and spatial characterizations.

**Figure 9:**
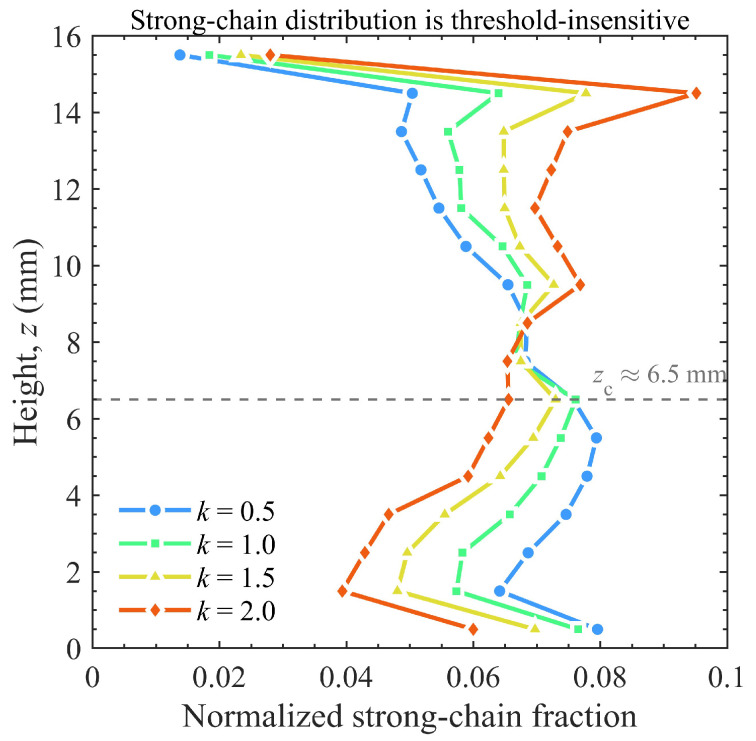
Normalized height distribution of strong force chains for four thresholds, k = 0.5, 1.0, 1.5 and 2.0 (strong contacts defined by Fn > k⟨Fn⟩). For every threshold, the strong-chain density rises across the transition (z_c_ ≈ 6.5 mm, dashed line) into the upper shear-active zone; the upper-zone concentration strengthens monotonically with k, confirming that the spatial organization is insensitive to the cutoff.

To confirm that this spatial organization is not an artifact of the strong/weak cutoff, the strong-chain height distribution was recomputed for four thresholds, k = 0.5, 1.0, 1.5 and 2.0 (strong contacts defined by Fn > k⟨Fn⟩; Figure 9). The strong-chain centroid remains in the upper zone for every threshold (z_strong_ = 7.06, 7.49, 7.98 and 8.52 mm, all above the transition height z_c_ ≈ 6.5 mm and above the all-contact centroid of 6.83 mm), and the upper-zone bias strengthens monotonically with k. The concentration of strong chains in the shear-active zone is therefore robust to—indeed, conservative under—the threshold selection rather than an artifact of the particular cutoff.

### 3.6. Effect of Sample Height

**Figure 10:**
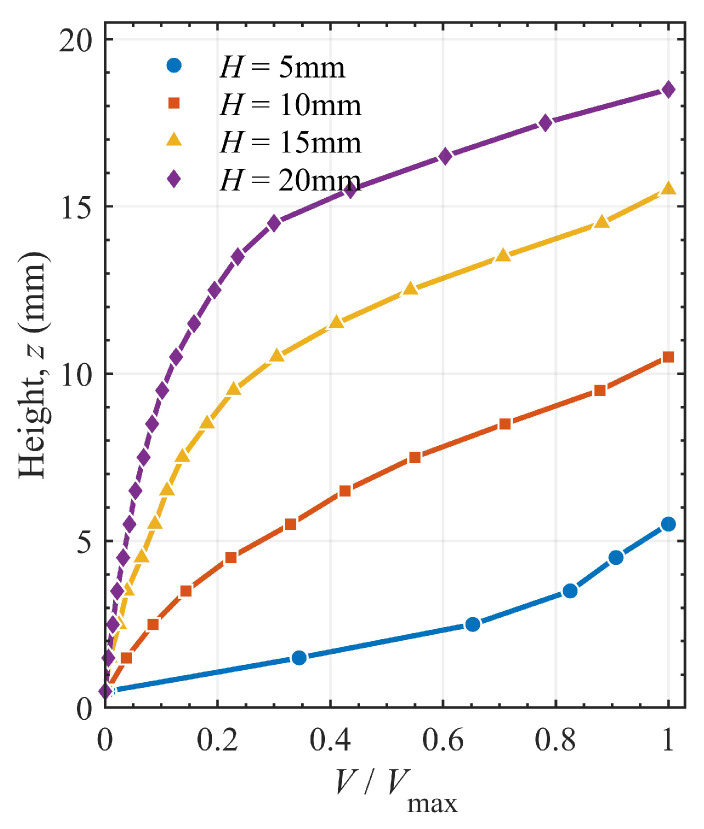
Steady-state tangential velocity profiles (averaged over 3–5 s) for sample heights H = 5, 10, 15 and 20 mm under identical shear conditions (σ = 1 kPa, v = 0.01 m/s). For tall samples (H = 15 and 20 mm), the near-boundary velocity decay collapses onto essentially the same curve, indicating a height-invariant shear-band thickness, whereas the thin sample (H = 5 mm) exhibits a qualitatively different, near-linear profile in which shear spans the entire sample; H = 10 mm is transitional.

To assess whether the localized-shear transition depends on the specific sample geometry, we repeated the simulation at four sample heights (H = 5, 10, 15 and 20 mm) under otherwise identical conditions (σ = 1 kPa, v = 0.01 m/s). The steady-state tangential velocity profiles are shown in Figure 10. For sufficiently tall samples (H ≳ 15 mm), the velocity decays away from the driven boundary over the same near-boundary length scale, so that the H = 15 and 20 mm profiles share an essentially height-invariant shear-band thickness. As the sample is thinned, the behaviour changes qualitatively: the H = 5 mm sample exhibits an almost linear profile in which shear spans the entire depth, with H = 10 mm representing a transitional case. The persistence of a fixed near-boundary shear band for tall samples, and its breakdown only when the sample becomes too thin to contain it, indicate that the localized-shear transition reported here is an intrinsic feature of the confined quasi-static regime rather than a consequence of the particular sample height.

## 4. Discussion

A central observation of this work is that four microstructural descriptors—tangential velocity, solid volume fraction, particle vertical mobility, and force-chain organization—undergo concurrent spatial transitions within a narrow height band centred near z ≈ 5–6 mm. The tangential velocity remains essentially zero below this elevation and rises exponentially above it (Figure 3a); φ stays tightly clustered at ≈ 0.50 with negligible time variability (Δφ ≲ 0.01) for z < 6 mm, then declines steadily toward ≈ 0.35 in the upper layers, with a marked broadening of the ±1σ envelope (Figure 4a); tracer particles originating above z_0_ ≈ 5–6 mm sustain velocities of several mm/s, persistent angular motion, and large vertical excursions, while those originating below remain effectively frozen (Figure 5); and the mean contact force begins to rise while the chain density begins to decrease at the same elevation (Figure 7b). The spatial co-location of these transitions indicates that the granular material self-organizes into two mechanically distinct strata: a quasi-rigid basal layer (z ≲ 5 mm) maintained by gravity-induced compaction, and an upper shear-active zone (z ≳ 6 mm) in which reduced confinement permits dilation, persistent contact reorganization, and selective load concentration on strong force chains. The emergence of such a sharp threshold at I ≈ 10^−3^ indicates that the competition between gravitational confinement and applied shear is resolved by kinematic localization rather than by distributed deformation [15,35,44]. To define this transition objectively, an onset height was computed for each descriptor as the point at which it departs by 10% of its full basal-to-upper change; the six descriptors cluster within ±1 mm of one another, giving z_c_ = 6.5 ± 1.0 mm (95% CI [5.5, 7.6] mm). The tight inter-descriptor agreement confirms that the stratification is a quantitatively robust, co-located feature rather than a visual inference, with the transition occupying a narrow band near z ≈ 5–7 mm. A conceptually analogous coexistence of a quasi-static base and a mobile sheared layer above has been described for collisional flows over erodible beds within the framework of extended kinetic theory [45,46]; the stratification reported here can be viewed as its quasi-static counterpart, with the governing physics shifting from collisional momentum transport to sustained frictional contacts.

The mechanism underlying this stratification can be traced to the contact force network. The exponential decay exponent β ≈ 0.45 corresponds to a heavy-tailed distribution in which forces well above the mean are common, consistent with persistent, long-lived chains in a quasi-static network [18,47]. In the upper layers, the reduced solid fraction (φ ≈ 0.35–0.38) implies a more open network in which load is carried by fewer, stronger chains, directly evidenced by the simultaneous rise in ⟨f⟩ and decline in N_c_ above z ≈ 5–6 mm (Figure 7b). In the basal region, by contrast, a dense network of comparatively uniform weak contacts distributes load across many bonds, accounting for the mechanical rigidity of this layer and its insensitivity to the imposed shear. The resulting μ_ss_ ≈ 0.78 therefore reflects the integrated effect of a heterogeneous contact network in which strong chains in the upper layers bear most of the shear stress [14,48]. Quantitatively, the value μ_ss_ ≈ 0.78 is consistent with the apparent friction coefficient μ = S/P measured in companion ring-shear DEM of the same material, which attains 0.4–0.6 in steady state (peaks ~0.75) and increases systematically toward lower confinement [24]. As the present case (σ = 1 kPa, P/P_0_ ≈ 0.02) lies at the extreme low-pressure end of that trend, the elevated μ_ss_ is expected; the value exceeds the canonical μ(I) quasi-static plateau for glass beads (μ_s_ ≈ 0.38) owing to enhanced geometric interlocking and reduced overburden stabilization at low confinement. These simulation results are consistent with our companion ring-shear experiments on the same glass-bead material [23]. Although those experiments were conducted at higher confinement and speed (σ = 100 kPa, v = 0.1 m/s), they exhibit the same near-universal velocity-profile geometry—an exponential-type decay from the driven boundary into a slowly creeping quasi-static region [23,35]—and a steady-state friction ratio τ/σ ≈ 0.6 of the same order as the simulated μ_ss_ ≈ 0.78 (cf. the quantitative comparison above). This agreement in profile geometry and friction magnitude provides experimental support for the present model.

This stratified picture has direct implications for the engineering characterization of granular materials at low overburden, where bulk-flow descriptors such as effective friction and apparent solid fraction are spatially varying fields rather than material constants. The kinematic threshold near z ≈ 5–6 mm implies that only an upper layer of finite depth participates in the imposed deformation, so characterization protocols that average over the full bed depth will systematically underestimate the local intensity of shear, dilation, and force-chain heterogeneity in the active zone. The exponential force distribution with β ≈ 0.45 provides a compact statistical descriptor of this heterogeneity, suitable for incorporation into material-characterization workflows alongside conventional bulk metrics.

From a constitutive modelling perspective, the stabilized μ_ss_ ≈ 0.78 supplies a calibration point for the quasi-static limit μ_s_ of the μ(I) framework, where direct DEM data are sparse [5,8]. The strong stratification documented here, however, also exposes a limitation of any local μ(I) closure in this regime: with deformation confined to an upper layer of finite depth and the basal region effectively frozen, a single bulk-averaged inertial number cannot capture the coexistence of quasi-rigid and shear-active strata within the same sample. Non-local frameworks incorporating spatial cooperativity between regions of differing plastic activity [20,21,22,49,50], and continuum models that treat granular temperature, packing fraction, and force-distribution heterogeneity as coupled fields, are therefore better positioned to reproduce the observed stratification. The kinematic threshold at z ≈ 5–6 mm, the height-dependent velocity gradient, the φ(z) profile with its basal plateau and upper-zone variability, and the exponential force distribution with β ≈ 0.45 together provide specific calibration and validation targets for such frameworks at the lower boundary of the dense flow regime.

## 5. Conclusions

Three-dimensional DEM simulations of dense annular shear at σ = 1 kPa and v = 0.01 m/s (I ≈ 1.06 × 10^−3^) reveal that the granular material self-organizes into two mechanically distinct strata under low-confinement quasi-static conditions: a quasi-rigid basal layer (z ≲ 5 mm; φ ≈ 0.50, negligible particle mobility, homogeneous weak-contact network) and an upper shear-active zone (z ≳ 6 mm; φ decreasing toward ≈ 0.35, persistent angular and vertical motion, selective concentration of strong force chains). The transitions in tangential velocity, solid-fraction temporal variability, particle mobility, and force-chain organization all co-locate within a narrow height band near z ≈ 5–6 mm, demonstrating that these four microstructural descriptors are not independent but spatially coupled manifestations of a single stratified state. The macroscopic friction coefficient stabilizes at μ_ss_ ≈ 0.78, and the contact force distribution exhibits a heavy-tailed exponential decay with β ≈ 0.45, with strong force chains concentrated selectively in the upper shear-active zone. The quantitative profiles reported here provide benchmarks both for the engineering characterization of granular materials operating at low overburden and for constitutive model development targeting the quasi-static limit of the dense flow regime. The present analysis is confined to a single representative operating point (σ = 1 kPa, v = 0.01 m/s); a systematic parametric study of the confining stress, shear velocity, interparticle friction, and particle size—to establish the universality of the z ≈ 5–6 mm transition—is the subject of ongoing work.

## Figures and Tables

**Table 1:** DEM model parameters.

Parameter	Symbol	Value
Particle density	ρ	2500 kg/m^3^
Shear modulus	GPa	72
Poisson’s ratio	ν	0.2
Restitution coefficient	e	0.938
Particle–particle sliding friction	μ_pp_	0.9
Particle–particle rolling friction	μ_r_	0.1
Particle–wall sliding friction	μ_pw_	0.6
Particle–wall rolling friction	μ_rw_	0.1

## Data Availability

The raw data supporting the conclusions of this article will be made available by the authors on request.
